# Supervised and Individualized Lifestyle Medicine Therapy of a Patient after Myocardial Infarction—Case Study

**DOI:** 10.3390/jcdd9060177

**Published:** 2022-06-01

**Authors:** Márton Dvorák, Ilona Sztancsik, László Babai, Miklós Tóth, Pongrác Ács

**Affiliations:** 1 Department of Health Sciences and Sport Medicine, Hungarian University of Sports Science, 1123 Budapest, Hungary; tothmik1@hotmail.com; 2 Kardioközpont, 1015 Budapest, Hungary; sztanmed@gmail.com (I.S.); efkbabai@uni-miskolc.hu (L.B.); 3 Faculty of Health Sciences, University of Miskolc, 3515 Miskolc, Hungary; 4 Institute of Physiotherapy and Sport Science, Faculty of Health Science, University of Pécs, 7621 Pécs, Hungary; pongrac.acs@etk.pte.hu; 5 Szentágothai Research Centre, University of Pécs, 7624 Pécs, Hungary

**Keywords:** lifestyle medicine, myocardial infarction, heart disease, exercise, rehabilitation

## Abstract

Background: In the rehabilitation of patients with cardiovascular disease (CVD) and after myocardial infarction (MI), lifestyle modifications—exercise and nutritional therapy, smoking cessation, stress management—are essential and have a major, long-term impact on the overall health of patients. Methods: After MI and acute primary care, a lifestyle medicine team (medical doctors, dietitian, exercise physiologist) supervised the therapy of a 40 year-old male patient for 12 months. The program included assessments, regular medical controls, personalized diet, and exercise therapy monitored by a heart rate watch. Results: Gradual and continuous weight loss, major reduction in medication and significant improvement in fitness level, blood glucose level, and cardiac function were measured after the program. Due to these positive changes, the patient’s overall health improved to an even better level than before his MI. Conclusion: The results of this program highlight the benefits and importance of the personalized therapy and the lifestyle medicine team approach in the rehabilitation of CVD patients. Individualized and supervised lifestyle therapy should be part of the primary hospital care of CVD patients lead by medical doctors and supported by other health care providers.

## 1. Introduction

Cardiovascular disease (CVD) is not only the most common cause of mortality, but its different levels impair the quality of life and lead to other comorbidities (such as type II diabetes, non-alcoholic fatty liver disease (NAFLD)) and CVD events (such as stroke, heart attack). In Europe, CVD accounts for 45% of all deaths [1]. A healthy lifestyle—especially regular physical activity and proper nutrition—should play a primary role in prevention and also in treatment and rehabilitation for CVD patients [2].

In Hungary, more than 16,000 patients get myocardial infarction (MI) every year [3] and it has one of the highest mortality statistics in Europe [1]. However, just 7.7% of them participated in the voluntary 3-week rehabilitation program in major heart hospitals [4] which is far from the participation, for example, in France (23%), the UK (29%), or the US (35%) [5]. Although in these institutes regular exercises and nutrition therapy are parts of the treatment, patients will not be monitored after completing the program, they will only have to return for another 3-week program a year later.

However, for a continuous and life-long program, patients need more professional support. Tailoring exercise and nutritional prescription according to the patient’s characteristics is necessary to optimize the effects of the therapy and improve the adherence [6]. Telemedicine solutions such as smartwatches are able to increase both supervision and motivation factors [7].

In the beginning and through the program, motivation is also important for the adherence and the effective changes in lifestyle behaviors. However, the formation of a chronic illness (such as high blood pressure, high cholesterol, diabetes) is not enough to change lifestyle habits, but a CVD event (stroke, MI) can trigger internal motivation or cause depression [8] at the same time. The internal motivators (which really matter for change) can be very diverse: awakening to a rapidly deteriorating state of health, fear of death, or even the birth of a first grandson or fulfilling a life dream such as competing in rally races. These internal motivators need to be supported by the lifestyle medical team not only on a professional but also empathetic approach in order to truly deliver a functioning, lifelong lifestyle change program to patients [9]. Patients are more likely to follow prescribed medical therapies if they understand the rationale for the treatment and have a collaborative relationship with medical professionals. Motivation and information about healthy lifestyle can lead to behavior change which is the key element of lifestyle medicine and should be well structured [10].

Thus, this study aims to report the 12 months of work of an optimal lifestyle medicine team, using a prescription of personalized exercise and nutritional therapy with medical supervision as cardiac rehabilitation to improve quality of life of a patient underwent non-ST segment elevation myocardial infarction (NSTEMI).

## 2. Detailed Case Description

In the present case study, a 40 year-old male patient was hospitalized for unstable angina without electrocardiogram (ECG) deviation but increased Troponin I (7.4510 µg/L), CRP (8.6 mg/L), and Creatine Kinase (627 U/L) levels. Echocardiography showed lower diastolic function (E/A ratio 0.78) and average ejection function (EF 58%), and a blood test showed elevated blood glucose level (8.1 mmol/L) (Table 1). Because of the formed NSTEMI, the coronarography showed subtotal occlusion in the left anterior descending (LAD) D1 artery and nonsignificant LAD stenosis. The deviation was found to be unsuitable for percutaneous coronary intervention (PCI) and the Heart Team suggested lifestyle medicine therapy.

Before this event, the patient (bodyweight (BW) 91.0 kg, body mass index (BMI) 29.7 kg/m^2^) lived an inactive, non-healthy lifestyle: he did not do exercises, smoked an average of 17 cigarettes a day, and usually worked a lot on his own business. He was treated for restless legs syndrome and hypercholesterinemia.

After the NSTEMI event, he stopped smoking and started the 3 weeks therapy in a public inpatient hospital for cardiac patients where baseline measurements were established. When it was finished, the patient still regularly felt chest pain and weakness and for more information he visited a private medical center where his supervised 12 months lifestyle therapy took with the support of a lifestyle medicine team. The leader of this team was a cardiologist who suggested developing a healthy lifestyle which included mainly regular physical activity, healthier nutrition habits, and stress management. Whereas the majority of the interactions between medical professionals and patients occurred with the medical doctor and exercise physiologist, many other health professionals were involved, depending on the needs of the patients, including diabetologist, neurologist, and hematologist. After the examinations with these medical doctors, the program leader cardiologist involved an exercise physiologist (EP) and a dietitian into the team and the individualized program was started.

**Figure 1:**
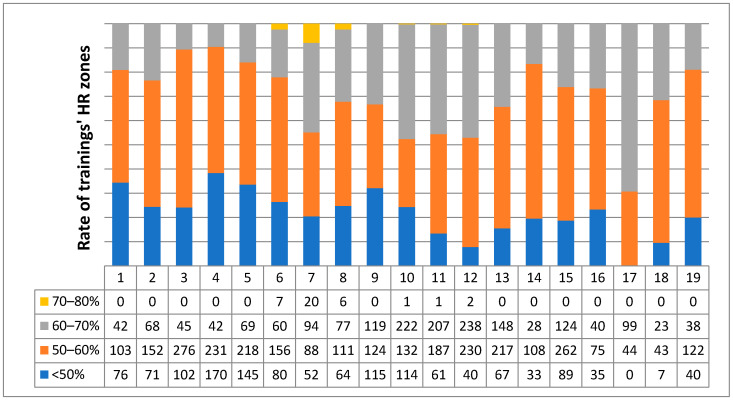
Trainings’ rate spent in different HR zones during second half of the program. Maximum HR (100%) was determined from the 220-age formula and intensity zones (here seen with different colors) were calculated from that HR for the patient. In the first row, numbers of the monitored weeks (1–19) are seen.

The EP performed an initial baseline fitness evaluation and designed an individualized exercise program. The program included one supervised personal training per week and home trainings (mainly indoor cycling, later badminton was allowed). The exercise protocol included principally cardio exercises (walking, cycling, rowing) in at first low (45–60% of maximal heart rate (MHR)) then moderate (60–70% of MHR) heart rate (HR) zones. In the personal trainings, strength exercises with small weights and moderate repetitions (10–15 reps) were also included. All of the trainings (weekly average 231 min in 8.85 occasion) were controlled by a heart rate monitor (brand Safako at first). Detailed exercise intensity data (Figure 1) were analyzed from week 24 when the heart rate monitor was changed to POLAR A300 (Kempele, Finland). During each personal training, the home workouts were also discussed and corrected by the EP.

The dietitian performed an evaluation and suggested an individualized nutrition plan which was mainly cardioprotective diet with increased whole grains, fruits, vegetables, low-fat dairy and meat products, vegetable oils, and decreased sugar products and salt. Because the patient followed this diet strictly and weight loss was continuous, more consultation with dietitian was not justified.

As a result of the continuous collaboration of the experts, the patient was provided with a complex program that improved his health through changes in habits in all areas of his life. This teamwork (of which the patient was also a part as an important factor of the program) greatly contributed to the results.

## 3. Results

After the 12 months lifestyle program, coronarography showed a moderate lesion on LAD and otherwise intact coronary arteries. Echocardiography showed wider left heart, good systolic left ventricular function, impaired relaxation, no visible regional wall movement disorder, and elevated mean arterial pressure. It was also seen that heart walls were contributing normally and E/A ratio increased significantly from 0.78 to 1 which can predict good diastolic function. No further deterioration in cardiac function was seen during the program. The patient had no symptoms as chest pain and weakness, his quality of life significantly increased.

Weight loss was gradual and continuous during the 12 months: decreased 14.0 kg (15.3% of the initial weight) and BMI decreased from 29.7 kg/m^2^ to 25.1 kg/m^2^. The fitness level of the patient measured by cycling ergometry with modified Bruce protocol increased significantly from 7.0 to 13.4 MET. Resting heart rate (RHR) measured by ECG decreased from 73 bpm to 66 bpm (Table 1) despite medication reduction. Average blood pressure was 97/63 mmHg during the program, but the medication was changed again due to dizziness and fatigue caused by too low blood pressure. After the 12 months, the medication changed significantly (Table 2) with the reduction of Atoris (statin), Coverex AS (ACE inhibitor), and cessation of Zyllt (anticoagulant), Amlopidin (calcium channel blocker), Frontin (antidepressant), and Erimexol (dopamine-agonist).

## 4. Discussion

The results of the complex lifestyle medicine program demonstrate that changing lifestyle behaviors is necessary for rehabilitation after NSTEMI. Although this finding is consistent with other studies [11,12], it shows the importance of a well-organized lifestyle medicine team with a dietitian, EP, and medical doctor as the leader of the team. The team was successful in encouraging the patient to adopt healthy behaviors to his (and his family’s) life. Teamwork, the unique expertise of the health care providers, and the supportive communication to the patient played a big role in the continuous improvement of his health and protected him from setbacks (such as restart smoking, stop doing physical activity or quit the whole program). The individualized program complied with the patient’s clinical and biological characteristics. The initial results include dramatic decrease in medication, significant improvements in fitness level, blood glucose levels, cardiac function, bodyweight, and BMI which put the patient in a significantly better state of health than before the MI. During the cardiological measurements (Echocardiography and ECG) fluctuating and not significant results were observed: Ao.asc., LV, AoVmax, TAPSE, PQ, and QRS decreased after 6 months and increased for the 12 months. The opposite trend was seen in DT and APSP. Based on these results, we conclude that the heart after the MI returned to normal state as a result of the program and no further deterioration occurred. However, we do not know these results before the MI.

Certainly, for this kind of results, the attitude of the patient is essential. His powerful initial motivator was clear from the start: he wanted to become healthier and compete in amateur rally races for which he needed a sports medical license. It was important for the providers to understand this motivator and build on it, because in a 12 month program there were many setbacks and weak moments, but this initial motivator factor with the support of the professionals helped the patient through the program. This coach approach, with an emphasis on connection and teamwork, is one factor that sets the lifestyle medicine intervention apart from traditional medical services [13]. The supportive family attitude was also important for success.

Although other research [14] has reported the opposite results, exercise interventions in health programs improve patients’ overall health and cardiac function [6,12]. There is also evidence that regular moderate exercise can reduce rehospitalizations among patients with stable coronary artery disease [15], especially if it is accompanied by smoking cessation and a Mediterranean diet [11]. These results confirm that rehabilitation of CVD patients with lifestyle medicine therapy is particularly important to improve the patient’s quality of life.

## 5. Conclusions

Individualized and supervised lifestyle modifications are essential tools in rehabilitation processes for CVD patients. However, these lifestyle medicine programs with monitoring medical care, exercise interventions, and nutrition consulting as a part of large-scale rehabilitation has several challenges. They require—among others—training facilities, professional exercise specialists and dietitians, online documentation platforms, and major financial resources. In order for such programs to become widespread and accessible to the local patients, they should be included in primary hospital care, for which efforts are already being made in Hungary.

It is not possible to know which part of the lifestyle change mainly caused the results, but we think this is not important either, because the program must cover all parts of the lifestyle in order for the patient to more effectively improve his own health. In Hungary, the acute medical care of patients with MI is as professional as in other European countries but for the aftercare, the compliance is significantly lower than in other countries [4]. These facts indicate the importance of long-term and personalized lifestyle medicine programs for cardiovascular patients.

Finally, we point out that, as this is a case report, the results presented here must be analyzed with caution. In this case, excellent results required a meeting between a well-prepared medical team and a motivated patient, which is rare and often not feasible. We know that a case report is only based on the hierarchical scale of scientific evidence. However, it shows what can be achievable under appropriate conditions in CVD rehabilitation.

## Figures and Tables

**Table 1:** Changes in physiological parameters.

Echocardiography	Baseline	Months 6	Months 12
Ao. asc. (mm)	38	29	31
LV (mm)	57 × 31	43 × 29	56 × 40
AoVmax (m/s)	1.43	1.1	1.2
LVOT VTI (cm)	24	19	18.8
E/A	0.78	0.88	1
DT (ms)	157	216	176
APSP (mmHg)	17 + 5	24 + 10	20 + 10
TAPSE (mm)	20	22	27
EF (%)	60	n/a	58
**Electrocardiography**			
HR (beat/min)	73	72	66
PQ (PR) (ms)	173	169	190
QRS (ms)	95	75	94
**Fitness level** (MET)	7.0	9.9	13.4
**Bodyweight** (kg)	91.0	80.5	77.0
**BMI** (kg/m^2^)	29.7	26.3	25.1
**Blood glucose** (mmol/L)	8.1	5.6	5.3

Ao. asc., ascending aorta; LV, left ventricle; LVOT VTI, left ventricular outflow tract velocity time; E/A ratio, diastolic function; DT, deceleration time; APSP, assisted peak systolic pressure; TAPSE, tricuspid annular plane systolic excursion; EF, ejection fraction; HR, heart rate; BMI, body mass index.

**Table 2:** Changes in medication.

	Baseline	Months 6	Months 12	Type of Drugs
**Medication**	100 mg Aspirin protect	100 mg Aspirin protect	100 mg Aspirin protect	Antiplatelet agent
	75 mg Zyllt	75 mg Zyllt		Anticoagulant
	80 mg Atoris	80 mg Atoris	40 mg Atoris	Statin
	1 × 2.5 mg, 1 × 1.25 mg Concor	1 × 2.5 mg, 1 × 1.25 mg Concor	5 mg Nebilet	Beta blockers
	10 mg Coverex AS	5 mg Coverex AS	2.5 mg Coverex AS	ACE inhibitor
	40 mg Pantoprazol	40 mg Nolpaza	40 mg Nolpaza	Proton pump inhibitor
	80 mg Adexor Prolong	80 mg Adexor Prolong	80 mg Adexor Prolong	Anti-ischemic agent
	2.5 mg Amlodipin			Calcium channel blocker
	2 × 0.125 mg, 1 × 0.25 mg Frontin	0.25 mg Frontin		Antidepressant
	26 mg Erimexol	26 mg Erimexol		Dopamine agonist

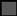
 Medication cessation. 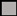 Medication reduction.

## Data Availability

All data generated and analyzed during this study are included in this manuscript.

## References

[1] Timmis A., Townsend N., Gale C., Grobbee R., Maniadakis N., Flather M., Wilkins E., Wright L., Vos R., Bax J. (2018). European Society of Cardiology: Cardiovascular Disease Statistics 2017. Eur. Heart J..

[2] Minich D.M., Bland J.S. (2013). Personalized lifestyle medicine: Relevance for nutrition and lifestyle recommendations. Sci. World J..

[3] Imre B., Andor S., Tímea C., István Á., Eszter S., Dóra E. (2019). Performance indicators of cardiac rehabilitation in Hungary. Orvosi Hetilap..

[4] Simon É., Bakai J., Dézsi C.A., Nagy L., Tóth L. (2017). Szívinfarktusos betegek akut és rehabilitációs ellátása Győr-Moson-Sopron és Vas megyében. Cardiol. Hung..

[5] Saito M., Ueshima K., Saito M., Iwasaka T., Daida H., Kohzuki M., Makita S., Adachi H., Yokoi H., Omiya K. (2014). Safety of exercise-based cardiac rehabilitation and exercise testing for cardiac patients in Japan-A nationwide survey. Circ. J..

[6] Passantino A., Dalla Vecchia L.A., Corrà U., Scalvini S., Pistono M., Bussotti M., Gambarin F.I., Scrutinio D., La Rovere M.T. (2021). The Future of Exercise-Based Cardiac Rehabilitation for Patients with Heart Failure. Front. Cardiovasc. Med..

[7] Ahuja N., Ozdalga E., Aaronson A. (2017). Integrating Mobile Fitness Trackers Into the Practice of Medicine. Am. J. Lifestyle Med..

[8] Ziegelstein R.C., Fauerbach J.A., Stevens S.S., Romanelli J., Richter D.P., Bush D.E. (2000). Patients With Depression Are Less Likely to Follow Recommendations to Reduce Cardiac Risk During Recovery From a Myocardial Infarction. Arch. Intern. Med..

[9] Mason P., Butler C. (2010). Health Behavior Change: A Guide for Practitioners.

[10] Stonerock G.L., Blumenthal J.A. (2017). Role of Counseling to Promote Adherence in Healthy Lifestyle Medicine: Strategies to Improve Exercise Adherence and Enhance Physical Activity. Prog. Cardiovasc. Dis..

[11] Booth J.N., Levitan E.B., Brown T.M., Farkouh M.E., Safford M.M., Muntner P. (2014). Effect of sustaining lifestyle modifications (nonsmoking, weight reduction, physical activity, and Mediterranean diet) after healing of myocardial infarction, percutaneous intervention, or coronary bypass (from the reasons for geographic and racial differences in stroke study). Am. J. Cardiol..

[12] Anderson L., Thompson D.R., Oldridge N., Zwisler A.D., Rees K., Martin N., Taylor R.S. (2016). Exercise-Based Cardiac Rehabilitation for Coronary Heart Disease. J. Am. Coll. Cardiol..

[13] Kent K., Johnson J.D., Simeon K., Frates E.P. (2016). Case Series in Lifestyle Medicine: A Team Approach to Behavior Changes. Am. J. Lifestyle Med..

[14] Moholdt T.T., Amundsen B.H., Rustad L.A., Wahba A., Løvø K.T., Gullikstad L.R., Bye A., Skogvoll E., Wisløff U., Slørdahl S.A. (2009). Aerobic interval training versus continuous moderate exercise after coronary artery bypass surgery: A randomized study of cardiovascular effects and quality of life. Am. Heart J..

[15] Vasankari V., Halonen J., Vasankari T., Anttila V., Airaksinen J., Sievänen H., Hartikainen J. (2021). Physical activity and sedentary behaviour in secondary prevention of coronary artery disease: A review. Am. J. Prev. Cardiol..

